# Adulthood cognitive trajectories over 26 years and brain health at 70 years of age: findings from the 1946 British Birth Cohort

**DOI:** 10.1016/j.neurobiolaging.2022.10.003

**Published:** 2023-02

**Authors:** Sarah-Naomi James, Jennifer M. Nicholas, Kirsty Lu, Ashvini Keshavan, Christopher A. Lane, Thomas Parker, Sarah M. Buchanan, Sarah E. Keuss, Heidi Murray-Smith, Andrew Wong, David M. Cash, Ian B. Malone, Josephine Barnes, Carole H. Sudre, William Coath, Marc Modat, Sebastien Ourselin, Sebastian J. Crutch, Diana Kuh, Nick C. Fox, Jonathan M. Schott, Marcus Richards

**Affiliations:** a MRC Unit for Lifelong Health and Ageing at UCL, University College London, London, UK; b Dementia Research Centre, UCL Queen Square Institute of Neurology, University College London, London, UK; c Department of Medical Statistics, London School of Hygiene and Tropical Medicine, University of London, London, UK; d School of Biomedical Engineering and Imaging Sciences, King's College London, London, UK; e UK Dementia Research Institute at UCL, University College London, London, UK; f UK Dementia Research Institute Centre for Care Research and Technology, Imperial College London, UK; g Department of Medicine, Division of Brain Sciences, Imperial College London

**Keywords:** Cognitive decline, Cognition, Brain health, Amyloid, Brain volume, Life course

## Abstract

Few studies can address how adulthood cognitive trajectories relate to brain health in 70-year-olds. Participants (n = 468, 49% female) from the 1946 British birth cohort underwent 18F-Florbetapir PET/MRI. Cognitive function was measured in childhood (age 8 years) and across adulthood (ages 43, 53, 60–64 and 69 years) and was examined in relation to brain health markers of β-amyloid (Aβ) status, whole brain and hippocampal volume, and white matter hyperintensity volume (WMHV). Taking into account key contributors of adult cognitive decline including childhood cognition, those with greater Aβ and WMHV at age 70 years had greater decline in word-list learning memory in the preceding 26 years, particularly after age 60. In contrast, those with smaller whole brain and hippocampal volume at age 70 years had greater decline in processing search speed, subtly manifest from age 50 years. Subtle changes in memory and processing speed spanning 26 years of adulthood were associated with markers of brain health at 70 years of age, consistent with detectable prodromal cognitive effects in early older age.

## 1 Background

There is growing evidence that age-related cognitive decline begins in mid adulthood, and is shaped by influences earlier in the life course, such as childhood cognitive ability, socioeconomic deprivation, and education (Davis et al., 2017). However, it is not yet fully established how cognitive decline throughout mid adulthood-to-later-life may correlate with brain health markers of early cerebral pathology. Several studies report associations between faster cognitive function and decline across adulthood, particularly in domains of episodic memory and processing speed, and worse brain health in cognitively-healthy older participants, such as greater Aβ load (Baker et al., 2017; Resnick et al., 2010), greater white matter hyperintensities (Kloppenborg et al., 2014) and smaller brain and hippocampal volume (Mungas et al., 2005). There is also evidence of the important role of *APOE-ɛ4* allele in cognitive ageing, a key risk factor for AD (Lim et al., 2015; Mormino et al., 2014; O'Donoghue et al., 2018). However, previous studies investigating the link between cognitive ageing and brain health often have a lack of repeated cognitive measures spanning across mid-and-later life, limiting the ability to disentangle prodromal cognitive decline from premorbid cognitive function; a lack of cognitive measures sufficiently sensitive to detect subtle age-related cognitive change; small sample sizes; and inadequate control for confounding of later-life cognition, such as childhood cognition, socioeconomic deprivation and education.

Drawing from a well-characterized population-based study, the longest running birth cohort of people born in the same week (the 1946 British Birth cohort), we can expand on previous studies by thoroughly characterizing individual changes in cognitive function over 26 years throughout mid-to-late life, taking into account key premorbid contributors of adult cognitive performance including childhood cognitive performance. First, we investigate how cognitive decline across 26 years, in the domains of verbal episodic memory and processing speed, vary by indices of brain health at age 70 years (including beta-amyloid (Aβ) deposition; whole brain volume; hippocampal volume; and white matter hyperintensity volume (WMHV) load). Second, we investigate the extent to which these relationships are independent or additive with other concurrent brain health measures, and the role of sex and *APOE-ε4* status in these relationships. Third, we investigate whether there are particular relevant periods of cognitive change throughout mid-to-later life that are linked with brain health at 70 years of age.

## 2 Methods

### 2.1 Participants

Study participants were from Insight 46, a neuroimaging sub-study of the National Survey of Health and Development (NSHD). NSHD initially comprised of 5362 individuals born throughout mainland Britain in 1 week in March 1946 (Kuh et al., 2016) with follow-up including over 24 contacts since birth, spanning over 69 years of follow-up. Eligibility criteria (Lane et al., 2017) and an overview of recruitment for Insight 46 (James et al., 2018) are outlined elsewhere. In brief, participants were eligible for recruitment if they met the criteria of having a defined set of life course data available and expressed willingness to come to a London-based neuroimaging and clinic visit (recruitment overview outlined in A._1_ (James et al., 2018)). Of the 841 participants invited, 502 (60%) attended the clinic at University College London with a detailed clinical, cognitive, and brain imaging protocol. Higher educational attainment, non-manual socio-economic position (SEP), higher cognition, being a non-smoker andbetter self-rating health were predictors of recruitment into the neuroimaging sub-study, but there were no differences between sex and APOE-e4 status (James et al., 2018) (further information and flow chart in A._1_). Ethical approval for the neuroscience sub-study was granted by the National Research Ethics Service (NRES) Committee London (14/LO/1173). All participants gave written informed consent.

### 2.2 Procedures

Imaging was performed on a single Biograph mMR 3T PET/MRI scanner (Siemens Healthcare, Erlangen), with simultaneous acquisition of dynamic PET/MR data including volumetric (1.1mm isotropic) T1 and FLAIR sequences; the full imaging protocol has been described previously (Lane et al., 2017). MRI sequences included: 3-dimensional T1-weighted MPRAGE images (voxel size 1.1 × 1.1 × 1.1 mm^3^ isotropic; TE/TR = 2.92/2000, total time = 5 minutes 6 seconds) and 3-dimensional FLAIR images using an IR-SPACE acquisition scheme (voxel size 1.1 × 1.1 × 1.1 mm^3^ isotropic; TE/TR = 402/5000, total time = 6 minutes 27 seconds) (Lane et al., 2017). All MRI data were preprocessed for gradwarp, image inhomogeneity and underwent a detailed quality control process by trained assessors, in line with protocols developed for commercial trials, who assess motion and coverage (Lane et al., 2017).

#### 2.2.1 Amyloid PET

PET data were acquired continuously in list-mode, during and following injection of 370 MBq florbetapir F18 (Amyvid). Aβ burden was assessed over a 10-minute period, ∼50 minutes after injection. PET data were processed using an automated in-house processing pipeline including pseudo-CT attenuation correction (Lane et al., 2017). Global standardized uptake value ratio (SUVR) was calculated from cortical regions of interest (ROIs), normalized to eroded subcortical white matter. Aβ status (+/-) was determined using a Gaussian mixture model applied to SUVR values, taking the 99th percentile of the lower (Aβ-) Gaussian as the cut-point (0.6104)(Lane et al., 2019).

#### 2.2.2 Brain volume

Volumetric T1-weighted images underwent visual QC before processing using automated pipelines for whole-brain segmentation using Multi-Atlas Propagation and Segmentation (Lane et al., 2017; Leung et al., 2011).

#### 2.2.3 Hippocampal volume

Volumetric T1-weighted images underwent visual QC before processing using automated pipelines for hippocampal region segmentation using Similarity and Truth Estimation for Propagated segmentations followed by manual checking and approprite editing (Jorge Cardoso et al., 2013).

#### 2.2.4 White matter hyperintensity volume (WMHV) segmentation

A validated, unsupervised, automated algorithm, Bayesian Model Selection (BaMoS) (Sudre et al., 2015), was used to segment white matter hyperintensities jointly from 3D T1 and FLAIR images, followed by visual quality control, generating a global white matter hyperintensity volume (WMHV) including subcortical grey matter but excluding infratentorial regions.

Models including brain volume were all adjusted for total intracranial volume (TIV), as calculated using SPM12 (Malone et al., 2015). More detail for derivation for all imaging measures are provided in A._1_.

### 2.3 Adulthood cognitive measures

Four assessments measuring a range of health metrics were carried out throughout mid-to-later adulthood (ages 43, 53, 60-64 and 69 years), including a cognitive assessment administered by research nurses according to a standardized protocol (Wadsworth et al., 2006). Thetwo main cognitive tests, word learning test (WLT) and visual search speed, were first chosen to be implemented in 1989 as they represented 2 fundamental aspects of fluid ability; speed of processing (visual search speed [Salthouse, 2000]) and verbal memory (wordlist learning test, adapted from the California Verbal Learning test (Elwood, 1995)). These tests were chosen as they were sensitive to age and morbidity-associated decline and suitable for large scale population-based cognitive testing given that they can easily be administered, display a good distribution r of ability in the population, have limited ceiling or floor effects, and are able to tdiscriminate changes over time (Houx et al., 2002).

The (WLT was assessed by recall of a 15-item word list where participants were shown each word for 2 seconds (Davis et al., 2017). Participants were then immediately asked to recall these words within 1 minute (immediate recall). The total number of words correctly recalled over 3 identical trials was summed to provide an overall score for WLT (maximum 45). Two word lists were alternated between study visits to minimize practice effects. Notably, the first WLT assessment in 1989 only assessed immediate free recall and did not assess delayed recall as is commonly used with this test. The neurocognitive test battery was repeated in the same manner for the same individuals in subsequent testing waves (in 1999, 2009 and 2015) to keep consistency of measures and ability to measure intra-individual changes in these measures. However, there was an additional fourth prompt for delayed recall of the word list in the 2009 testing wave, whereby participants were asked to recall as many words as possible around 10 minutes after the initial presentation and after another cognitive task. A._2_ outlines a sensitivity analysis that shows there were no differences in the pattern of associations with brain health measures depending on whether the immediate or delayed measure of WLT was used at this timepoint.

Processing search speed was assessed by a visual search task, where participants were required to cross out the letters P and W, randomly embedded within a page of other letters, as quickly and accurately as possible, within 1 minute (Davis et al., 2017). Search speed was represented by the position reached at the end of this interval (maximum 600).

#### 2.3.1 Covariables

Based on previous studies of predictors of later-life cognition and cognitive ageing (Hatch et al., 2007; Lu et al., 2019a; Opdebeeck et al., 2016; Richards et al., 2019, 2014, 2001), the following variables were treated as potential confounders: sex, childhood cognition, childhood and adulthood socioeconomic position (SEP) and educational attainment. Given that participants were born in the same week and subsequently were very closely aged during cognitive assessments , only age at the time of the scan was treated as a potential confounder, although notably there is still a narrow age range for the scan (69.2–71.9 years).

Childhood cognition was adjusted since this was previously shown to be associated with the intercept and slope of decline in the cognitive tests (Davis et al., 2017; Richards, 2001). Childhood cognition was measured at age 8 using tests of reading comprehension, pronunciation, vocabulary and non-verbal reasoning (Pigeon, 1964)**.** Scores from each test were standardized to the tested sample at the time. Where data were missing, z-scores from assessments at age 11 or age 15 years were substituted. Sensitivity analyses were conducted, using WLT trajectories and Aβ as an example,  to investigate if the results differed depending on how childhood cognition was operationalized as a covariate.. Models were re-run using childhood cognition i) with and without re-standardizing to the analytical sample; ii) with and without imputing missing cognitive scores with standardized scores ascertained at ages 11 and 15 years. The results revealed very little difference in the pattern of effects (A._3_).

Childhood SEP was derived from occupational class of the father; adulthood SEP was derived from participants’ own occupation at 53 years, or earlier than this if information was missing. SEP was coded according to the UK Registrar General's Standard's Occupational Classification and further categorized into manual (skilled manual, semi-skilled manual and unskilled) or non-manual (professional, intermediate, skilled non-manual) professions. The highest educational attainment by 26 years was classified according to the Burnham scale and grouped into the following: no qualification, up to GCE (taken ∼age 16), and advanced level and above (‘A’ level, or degree or equivalent, taken after age 16).

Genotyping of the 2 SNPs, rs439358 and rs7412, used to determine *APOE* genotype was conducted at LGC, Hoddesdon UK. Individuals were categorized as *APOE*-ε4 homozygous, heterozygous or non-carriers. Mental health affective symptoms at age 69 were assessed using the 28-item General Health Questionnaire (GHQ-28)(Goldberg et al., 1997; Goldberg and Hillier, 1979). In line with previous studies (Hatch et al., 2009), a validated threshold of scoring greater than 4 on the GHQ-28 indicated a ‘case-level’ affective mental health problem.

### 2.4 Statistical analyses

#### 2.4.1 Analytical sample

Participants had to take part in the neuroimaging sub-study at age ∼70 years, pass the quality control for Aβ-PET or MR imaging, and have at least two follow-up cognitive assessments out at four throughout adulthood. There was limited missing data for cognitive and covariate measures across adulthood, missing data ranged from 0% to 6% (Table 1); 86% had complete data across all four cognitive assessments. Analyses were conducted using Stata version 15.1 and R version 3.5.1.

**Table 1:** Characteristics of participants

	All	n	% missing
Max n for analysis	468		
**Characteristics**			
Female (n, %)	229 (49%)	468	0.0
Age of years at scanning (mean, sd)	70.7 (0.7)	468	0.0
*Educational attainment*		468	0.0
None (n, %)	74 (16%)		
Up to GCE (up to age 16) (n, %)	140 (30%)		
A-level and above (age 16 and above) (n, %)	254 (54%)	468	0.0
*Child SEP*			
Non-manual (n, %)	267 (57%)	464	0.2
*Adult SEP*			
Non-manual (n, %)	397 (85%)	468	0.0
*APOE-ε4 status*			
No *ε4* (n, %)	327 (70%)	466	0.1
*ε4* Heterozygous (n, %)	127 (27%)		
*ε4* Homozygous (n, %)	12 (3%)		
Dementia	3 (0.6%)	468	
MCI (n, %)	7 (1%)	468	0.0
Affective symptoms at age 69 years (n, %)	32 (7%)	461	1.5
***Cognition***			
Childhood cognition (mean, sd)	0.4 (0.7)	468	0.0
Word learning test at age 43 years (mean, sd)	26.9 (5.6)	439	6.2
Word learning test at age 53 years (mean, sd)	26.3 (5.8)	459	1.9
Word learning test at age 60–64 years (mean, sd)	25.8 (5.6)	468	0.0
Word learning test at age 69 years (mean, sd)	23.7 (5.7)	457	2.4
Letter processing speed at age 43 years (mean, sd)	344.6 (71.6)	443	5.3
Letter processing speed at age 53 years (mean, sd)	288.1 (69.7)	456	2.6
Letter processing speed at age 60–64 years (mean, sd)	272.6 (65.5)	468	0.0
Letter processing speed at age 69 years (mean, sd)	266.8 (70.5)	456	2.6
***Neuroimaging variables, age 70 years***			
Aβ status (n, %)	86 (19%)	460	1.7
Standardized Uptake Value Ratio (mean, sd)	0.6 (0.07)	460	1.7
Hippocampal volume (mL) (mean, sd)	3.1 (0.3)	468	0.0
TIV-adjusted hippocampal volume (mL) (mean, sd, range)	0.03 (0.3)	468	0.0
Whole brain volume (mL) (mean, sd)	1100 (99)	468	0.0
TIV-adjusted whole brain volume (mL) (mean, sd)	0.05 (45.7)	468	0.0
White matter hyperintensity volume (mL) (mean, sd)	5.1 (5.4)	455	2.8
TIV-adjusted white matter hyperintensity volume (mL) (mean, sd)	0.01 (1)	455	2.8

Key: Aβ+, amyloid positivity; SEP, socioeconomic position; MCI, mild cognitive impairment; TIV, total intracranial volume.

#### 2.4.2 Cognitive trajectories over 26 years and brain health at age 70

Mixed effect regression models were applied to investigate how brain health at age 70 years, including measures of Aβ status (dichotomous); level of whole brain volume (continuous); level of hippocampal volume (continuous); and amount of WMHV (continuous), was linked to preceding cognitive decline across 26 years of adulthood (from age 43 to 69 years). Repeated performance of WLT and search speed at ages 43, 53, 60–64 and 69 years served as the longitudinal cognitive variables. A quadratic term for cognitive change over time was used as previously demonstrated, and models were fitted with random intercept and slopes with unstructured covariance matrices. Interactions between time (as linear and quadratic terms) and each brain health measure were tested seperately; a significant interaction indicated that decline in cognitive function over time differed by differing levels of the brain health measure of interest. Model fit was compared by selecting the lowest Bayesian Information Criterion (BIC). All models were adjusted for age at scan (spanning two years), and known important premorbid predictors of cognitive ageing including sex, childhood cognition, childhood and adulthood SEP and educational attainment.

#### 2.4.3 Role of concurrent brain health measures

To reduce multiple testing, only the cognitive trajectories and brain health measures that were significant in initial models were selected for further testing (A._5_).

To assess the independence of associations between cognitive trajectories and specific brain measures, significant models were re-run mutually adjusting for other measured brain health measures (Aβ, brain, hippocampal and WMH volume). To assess the potential effect-modifying nature of other brain health measures on cognitive trajectories, we added interaction terms between brain health measures with time. We additionally followed the approach employed by Jack et al and Bilgel et al. (Bilgel et al., 2018a; Jack et al., 2013) to create groups indicating those with evidence of amyloidosis (Aβ+) and small hippocampal volume, to investigate the extent to which cognitive trajectories vary in those with evidence of a combination of early pathological measures associated with Alzheimer's disease. In line with other studies, adjusted hippocampal volume (residual hippocampal volume from the hippocampal volume that would be expected at a given intracranial volume) at the 10th percentile or lower were categorized as the smallest hippocampal group (H+). This corresponded with a cut-off value of -0.4 cm^3^ and lower (n = 45). This is notably less than the -0.7 cm^3^ cut-off in Jack et al. 2012 which was derived in an older-age sample (mean age 78). Those who were Aβ+ were categorized as amyloid positive (A+, n = 86). Four levels of group status were subsequently derived: (1) A-H –(n = 341), (2) A+H-(N = 74), (3) A-H+(N = 33), (4) A+H+ (n = 12). Similarly defined mixed effect regression models were then applied to investigate how decline in adulthood cognitive function varied by amyloid and smaller hippocampal volume group status (categorical variable with 4 levels).

#### 2.4.4 Further adjustments and sensitivity analyses

We tested whether the relationships between the cognitive trajectories and brain measures differed by sex and APOE-*ε4* by adding interactions with time.Models were subsequently stratified if interactions were statistically significant (A._4_). If interactions were not significant, models were re-run adjusting for *APOE-ε4* status to assess the potential contributing nature. Models were re-run adjusting for affective mental health problems at the time of cognitive assessment to assess the potential impact of affective mood on cognitive performance; and re-run excluding those who met criteria for dementia (n = 3) or MCI at the time of the scan (n = 7) to explore if those with cognitive impairment were driving the associations. In line with our previously published papers (Lu et al., 2019b) and based on published criteria (Petersen et al., 2013), dementia was determined by expert consensus, informed by assessments at age 69–71 including clinical history, informant history, Mini-Mental State Examination (score=>26) (Folstein et al., 1975) and cognitive performance (WMS-R Logical Memory test (Wechsler D., 1987) and WAIS-R Digit symbol substitution test (Wechsler D., 1981) MCI was determined as follows: (1) no clinical evidence of dementia; *and* (2) participant concern regarding cognition (memory or cognitive difficulties more than other people the same age, or if they reported that they would seek medical attention regarding their difficulties) or informant concern regarding the participant's cognition (AD8 score ≥2); *and* (3) objective evidence of either an amnestic (Logical Memory delayed recall ≥1.5 SD below the mean) or nonamnestic deficit (digit substitution score ≥1.5 SD below the mean).

#### 2.4.5 Cognitive change and brain health at age 70 years

WLT and search speed change across smaller time epochs, for the periods between ages 43 and 53; ages 53 and 60; and ages 60 and 69 years of age, were calculated using individuals with available data at all time-points, similarly to our previous work (Lane et al., 2019). Cognitive change, conditional on earlier measurements, was calculated as the residual from the regression of each cognitive measure on the earlier measure. Residuals represent declining change in cognition that differed from changes expected on average given the earlier cognitive performance. Residuals were standardized, allowing comparisons between periods. The relationship between cognitive conditional change variables as the exposure and brain health measures as the outcome were assessed individually with linear regression models. Model assumptions were checked with regression diagnostics and possible non-linear associations were explored by introducing a quadratic term to fully adjusted models.

## 3 Results

### 3.1 Characteristics

Of 502 individuals assessed, 471 completed the imaging protocol. Following imaging processing and quality control, n = 460 participants were available for Aβ analysis, n = 468 for whole brain and hippocampal volume analysis and n = 455 for white matter hyperintensity volume analyses (flow chart shown in A_1_). Participant characteristics are summarized in Table 1. The mean age was 70.7 (SD 0.7) years and 49% were female. 18% of the sample were Aβ+.

### 3.2 Cognitive trajectories in adulthood (between ages 43–69 years) and brain health at age 70 years

There was a significant interaction with time for amyloid status, and WMHV, for WLT decline (Table 2). Compared to those who were Aβ- at age 70 years, those who were Aβ+ had faster rates of WLT decline in the preceding 26 years (-0.06 more decline in WLT per year, Fig. 2A). Those with greater WMHV at age 70 years had faster WLT decline in the preceding 26 years (-0.02 more decline in WLT per year for every 1ml increase in WMHV, Fig. 2B).

**Table 2:** Longitudinal decline of word learning test (WLT) and search speed measures from 43-69 years of age, varies by brain health measured at age 70 (Aβ status, brain, hippocampal and white matter hyperintensity volume and amyloid-hippocampal group status).

	WLT decline			Search speed decline		
Term	B	*p*	*95% CI*	B	*p*	*95% CI*
**Amyloid status**						
Main effect of Aβ+ status on mean cognition	0.32	0.59	-0.86-,1.51	0.33	0.97	-15.01,15.67
Interaction effect of Aβ by time per year (linear)	**-0.06**	**0.02**	**-0.11,-0.01**	-0.15	0.63	-0.78,0.47
**Brain volume**						
Main effect of BV on mean cognition	0.00	0.92	-0.01,0.01	0.02	0.76	-0.11,0.15
Interaction effect of BV by time per year (linear)	0.00	0.26	-0.00,0.00	**0.01**	**0.01**	**0.00,0.01**
**Hippocampal volume**						
Main effect of HV on mean cognition	-0.59	0.42	-2.03,0.85	-15.07	0.13	-34.36,4.23
Interaction effect of HV by time per year (linear)	0.03	0.39	-0.04,0.09	**0.99**	**0.02**	**0.14,1.84**
**White matter hyperintensity volume**						
Main effect of group on mean cognition	0.03	0.89	-0.44,0.50	-2.25	0.46	-8.29,3.78
Interaction effect of WMHV by time per year (linear)	**-0.02**	**0.01**	**-0.04,-0.01**	-0.17	0.19	-0.41,0.08
**Amyloid (A)**-**hippocampalvolume (H) group status**						
Main effect of group on mean cognition	6.05	0.10		1.73	0.60	
Interaction effect of group by time per year (linear)	**15.03**	**<0.01**		5.80	0.10	
Interaction effect:A-H- vs A+N- with time	**-0.06**	**0.02**	**-0.11,-0.01**	-0.04	0.92	-0.71,0.64
Interaction effect:A-H- vs A-N+ with time	**-0.09**	**<0.01**	**-0.15,-0.03**	-0.65	0.11	-1.43,0.14
Interaction effect:A-H- vs A+N+ with time	-0.12	0.12	-0.29,0.05	-1.30	0.06	-2.64,0.04

All models adjust for sex, age at scan, childhood cognitive ability, childhood and adulthood SEP, educational attainment. Models for white matter hyperintensity volume, brain volume and hippocampal volume were additionally adjusted for total intracranial volume.

Note: *p* < 0.05 denoted in bold. Key: Aβ, amyloid positivity; BIC, Bayesian Information Criterion; BV, brain volume; CI, confidence interval; HV, hippocampal volume. WLT, word learning test; WMHV, white matter hyperintensity volume.

**Fig. 1:**
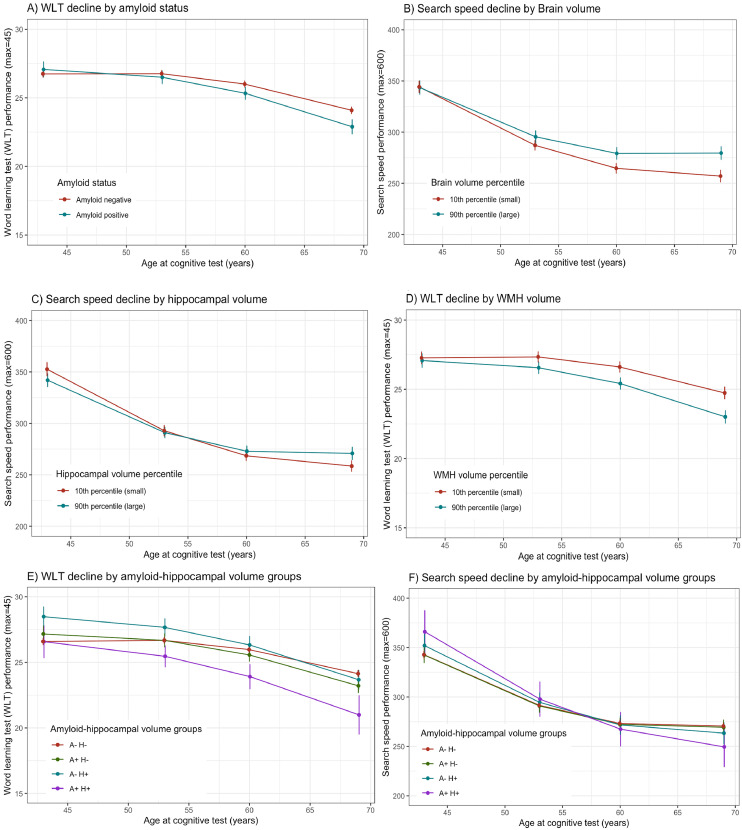
Associations betweencognitive trajectories of word learning test and search speed measures between 43 and 69 years of age that are associated with brain health measures at 70 years of age, including Aβ status (A) brain volume (B) hippocampal volume (C) white matter hyperintensity volume (D) and Aβ and hippocampal groups (E) at age 70. WLT=word learning test; A-H-=PET amyloid negative and hippocampal volume not in smallest decile (2) A+H-= PET amyloid positive and hippocampal volume not in smallest decile (3) A-H+= PET amyloid negative and hippocampal volume in smallest decile (4) A+H+= PET amyloid positive and hippocampal volume in smallest decile. NB: Continuous variables are illustrated as the 10th and 90th percentiles for graphical representation. Models adjust for sex, age at scan, childhood cognition, childhood and adulthood SEP and educational attainment. (For interpretation of the references to color in this figure legend, the reader is referred to the Web version of this article.)

There were significant interactions with brain and hippocampal volume at age 70 years for search speed trajectories; those with smaller brain volume, and smaller hippocampal volume, had faster search speed decline in the preceding 26 years (Table 2, Fig 1C and D): Search speed decline was -0.1 faster per year for every 10ml decrease in brain volume and -1 point faster per year for every 1ml decrease in hippocampal volume.

**Fig. 2:**
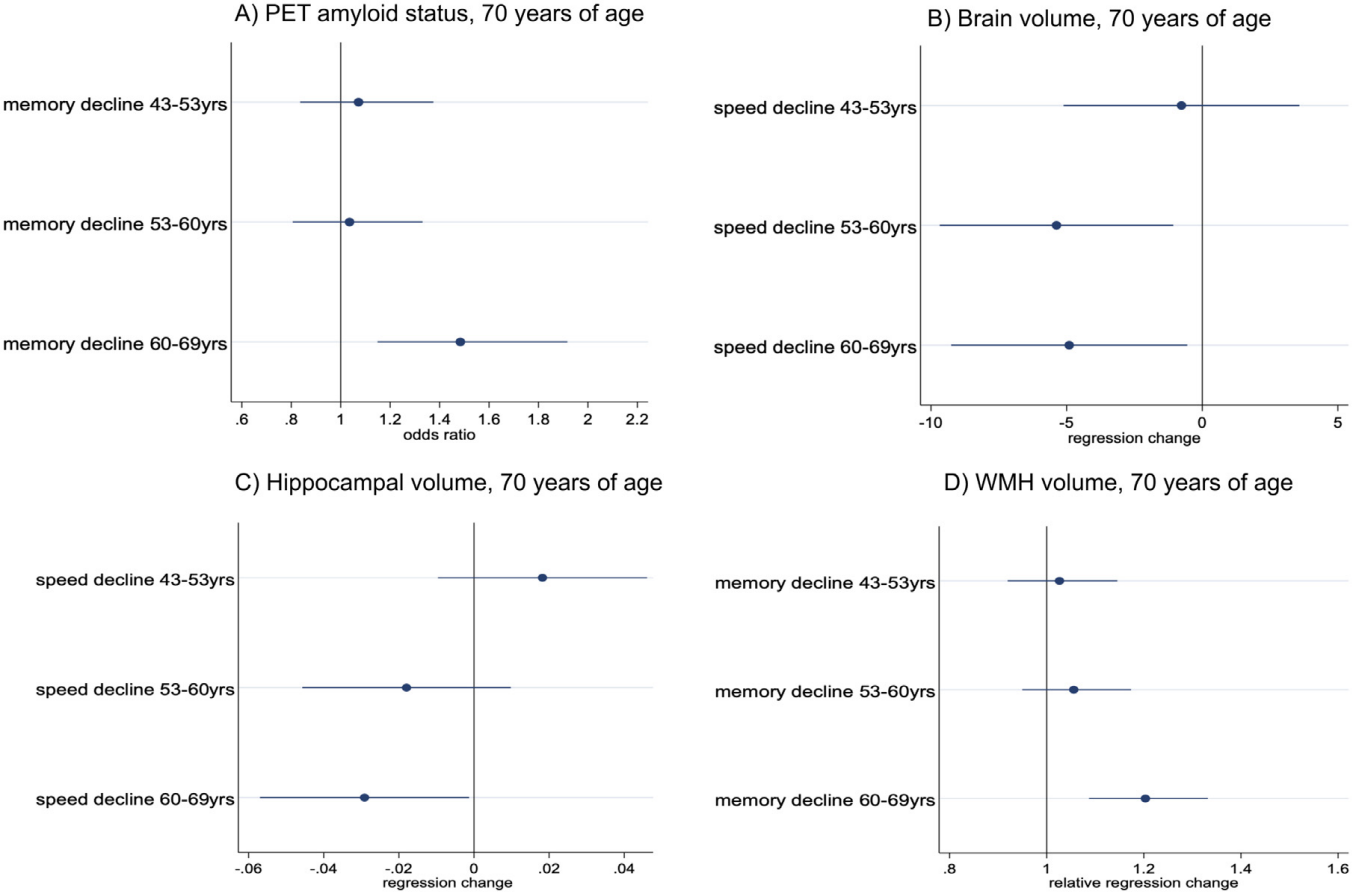
Associations of the relationships between specific cognitive change periods in adulthood and brain health measures at 70 years of age, including odds ratios for Aβ status (A), beta coefficient difference in brain volume (B) and hippocampal volume (C), and relative difference in white matter hyperintensity volume (D). Models adjust for sex, age at scan, childhood cognition, childhood and adult SEO and educational attainment. WMH, white matter hyperintensity volume; Yrs, years of age. Cognitive change, conditional on earlier measurements, was calculated as the residual from the regression of each cognitive measure on the earlier measure. Change unit represent declining change in cognition that differed from changes expected on average given the earlier cognition. Residuals were standardized, allowing comparisons between periods. (For interpretation of the references to color in this figure legend, the reader is referred to the Web version of this article.)

### 3.3 Role of concurrent brain health measures for cognitive trajectories

The effect sizes remained similar when brain health measures were mutually adjusted, and there was no evidence of interactions between brain health measures, demonstrating their independent relationship (A_.5_).

For Aβ-hippocampal volume group status, there were significant interactions for WLT decline over time (p=0.01). Compared to A-H- individuals, the rate of WLT was significantly faster in A+H- (-0.06 decline per year) and A-H+ individuals (-0.09 decline per year); and in the A+H+ individuals (-0.12 difference per year, although whilst the coefficient effect size was the largest, the difference was not significantly so, *p* = 0.12) (Table 2, Fig. 1E).

### 3.4 Adjustments and sensitivity analyses for cognitive trajectories

There was no evidence of sex or APOE-e4 interactions (A_.4_). The pattern of results for amyloid status attenuated with exclusion of those with dementia and MCI (n = 10, [-0.06, *p=*0.02] to [-0.03 *p* = 0.1]), but otherwise effect sizes remained similar after adjusting for APOE-ε4 and affective mental health problems at the time of cognitive assessment (A_.5_).

### 3.5 Cognitive change epochs and brain health at age 70

Looking at specific change periods, greater WLT memory decline than expected between ages 60 to 69 years was significantly associated with Aβ+ status and greater WMHV at age 70 (Table 3, Fig 2). Greater search speed decline between ages 53 to60 and 60 to69 years was associated with smaller brain volume at age 70; greater search speed decline between ages 60 to69 years was additionally associated with smaller hippocampal volume (Table 3).

**Table 3:** Conditional change of word learning test (WLT) and search speed measures from 43 to 69 years of age by varying levels of brain health at age 70 (Aβ status, brain, hippocampal and white matter hyperintensity volume (WMHV)).

Cognitive change periods	Coef	p	95% CI
**Amyloid status and WLT**^a^			
43–53 period of rate of change years	1.09	0.50	0.85,1.41
53–60–64 period of rate of change years	1.04	0.79	0.81,1.33
60/64–69 period of rate of change years	**1.48**	**<0.01**	**1.15,1.92**
**Brain volume and search speed decline**^b^			
43–53 years	-1.14	0.62	-5.69,3.41
53–60–64 years	**-5.79**	**0.01**	**-10.19,-1.39**
60/64–69 years	**-6.27**	**0.01**	**-10.79,-1.74**
**Hippocampal volume and search speed decline**^b^			
43–53 years	0.02	0.13	-0.01,0.05
53–60–64 years	-0.02	0.20	-0.05,0.01
60/64–69 years	**-0.03**	**0.04**	**-0.06,-0.01**
**WMHV and WLT**^c^			
43–53 years	1.03	0.64	0.92,1.15
53–60–64 years	1.06	0.32	0.95,1.17
60/64–69 years	**1.20**	**<0.01**	**1.09,11.33**

Cognitive change, conditional on earlier measurements, was calculated as the residual from the regression of each cognitive measure on the earlier measure. Change unit represent declining change in cognition that differed from changes expected on average given the earlier cognition. Residuals were standardized, allowing comparisons between periods.

a A logistic regression model was conducted for amyloid status where coefficients represent an odds ratio.

b Linear regression models were conducted for standardized brain volume and hippocampal volume, where coefficients represent a standardized change in standard deviation per unit change of cognition.

c A generalized linear model using the gamma distribution with log link conducted for white matter hyperintensity volume where coefficients represent a relative increase. Note: *p* < 0.05 denoted in bold. Key: Aβ, amyloid positivity; CI, confidence interval; WLT, word learning test; WMH, white matter hyperintensity volume.

## 4 Discussion

### 4.1 Main findings

In this well-characterized population-based longitudinal cohort of people born in the same week,we found detectable and subtle changes in cognition spanning 26 years of adulthood that were associated with various markers of brain health in the early 8th decade. Those with greater Aβ deposition and WMHV at 70 years of age had greater decline in word-list learning memory in the preceding 26 years, whilst those with smaller whole brain and hippocampal volume at age 70 years had greater decline in processing search speed. These associations remained when adjusted for known measures of premorbid later-life cognitive function (childhood cognition, childhood and adulthood SEP and educational attainment) and were independent of other brain health measures. Our findings suggest that subtle differences in memory and processing speed decline in mid to early older age are detectable and linked independently with various markers of brain health at age 70 years, including presumed markers of underlying pathophysiology associated with AD (Aβ), age-related changes and cerebral small vessel disease (WMHV).

Our finding that those with greater Aβ deposition at age 70 years had faster rates of preceding decline in memory, but not in processing speed, is in line with findings from a meta-analysis that demonstrates Aβ-related cognitive decline may manifest predominately in episodic and semantic memory measures in older age (Baker et al., 2017). However, a range of younger cohorts, often with participants below age 65, do not find associations between Aβ and episodic memory (Johnson et al., 2014; Mielke et al., 2016; Rodrigue et al., 2012). By studying individual-level cognitive trajectories across mid-to-later life in this study we were able to identify that decline in memory, particularly between ages 60–69 beyond the level expected given their prior cognition, was associated with greater Aβ load, suggesting that Aβ-associated cognitive change becomes manifest between ages 60–69. More generally, episodic memory decline is reported to be one of the first cognitive domains affected in AD (Bäckman et al., 2005; Bennett et al., 2006; Bilgel et al., 2017; Grober et al., 2008; Kirova et al., 2015). Although episodic tests vary, they encompass tasks which depend on the ability to organize serial and semantic information, and are thought particularly to require frontal and medial temporal function (Tromp et al., 2015). The WLT task we have used in this study relies on semantic processing to organize words during encoding and retrieval; similar versions have been associated with later-life functional ability (Gross et al., 2011).

The effects of Aβ and WMHV on WLT memory decline were independent and we found no evidence of an interactive effect at this time. This suggests that flevels of Aβ and cerebral small vessel disease present at age 70 years may act independently as additive, but not interactive, processes. However, potential interactions and synergistic effects may manifest as the cohort ages and the pathology burden increases, and for other un-measured pathology such as tau-protein and microstructural changes.

As Aβ is thought to be the earliest pathological change in AD with neurodegeneration effects downstream, as the cohort ages we may expect that WLT decline becomes associated with markers of neurodegeneration, indicating those in the more advanced stages of preclinical AD (Bilgel et al., 2018b). Indeed, our sensitivity analyses demonstrated that those who have evidence of greater amyloid deposition and have a disproportionately smaller hippocampal volume (10th decile) showed the fastest rate of decline in memory measures, which may reflect those with more advanced disease. Alternatively, a relevant study demonstrated that neurodegeneration (as indicated by small hippocampal volume) and amyloidosis have independent, as well as synergistic, negative effects on cognitive performance (Bilgel et al., 2018b), indicating that cognitive impairment may be amplified by co-existing pathology, although we did not find evidence of this.

Attenuation by ε4 or sex was not observed for any associations, despite APOEε4 being a strong predictor of Aβ deposition (Lu et al., 2019a), and memory (Rawle et al., 2018) in this cohort. However, ε4 may exert influence on cognition independent of AD-related pathology (Greenwood et al., 2005; O'Donoghue et al., 2018).

Our sample has fairly low levels of WMHV so it is striking that even in a low-risk population-based sample, higher WMHV at age 70 is associated with greater preceding WLT memory decline. WMHV burden is presumed cerebral small vessel disease and accumulates with age, but may be present even from the fifth decade (Moura et al., 2019). Our findings provide further evidence that WMHV are linked with worse cognitive performance, and may not be asymptomatic but instead may contribute to cognitive impairment (Garnier-Crussard et al., 2020). We were surprised to find that the level of WMHV at age 70 was not associated with processing speed decline given previous studies show WMHV is linked with most cognitive domains, including processing speed (Kloppenborg et al., 2014). However, as most studies are cross-sectional in design, or are in older populations, the ability to address the unique effects of WMHV on adulthood cognitive decline trajectories have been limited. In addition, in this study we did not look at regional WMHV, yet evidence shows that deep WMHV may be more strongly linked with processing speed (Bolandzadeh et al., 2012; Brugulat‐Serrat et al., 2019).

We found that those with smaller brain and hippocampal volume at 70 years had greater preceding search speed decline, independently of predictors of later-life cognition and other measured brain health. We further identified that decline in search speed, particularly between ages 53–60, and 60–69, beyond the level expected given their prior cognition, was associated with lower brain volume. We did not however find strong evidence for associations between rate of memory decline and brain and hippocampal volumes which was interesting. These findings are consistent with reports of reduced processing speed considered as a sensitive, yet non-specific proxy for brain ageing associated with global MRI brain volumes (Rabbitt et al., 2006).

### 4.2 Strengths and limitations

A major strength of this study is the availability of prospective assessed childhood measures of cognition which allow us to show that these findings of cognitive decline are independent of cognitive development and premorbid cognition. Having repeated measures of cognitive function domains across adulthood, in an age-homogenous and well characterized sample, further enable us to estimate sensitive cognitive decline age-effects over time (Gottesman et al., 2014). Our sample were scanned on the same PET-MRI machine enabling multiple measures of *in vivo* brain health and are at a prime age (age 70 (0.7)) to study brain health simultaneously; a relatively early risk period for cognitive impairment, but where neuropathology is accumulating (Villemagne et al., 2008).

Whilst the two cognitive tests assessed here represent fundamental aspects of fluid ability; speed of processing (visual search speed (Salthouse, 2000)) and verbal memory (word list test(Elwood, 1995)), which are sensitive to age and morbidity-associated decline (Davis et al., 2017), the longitudinal cognitive test battery did not include measures of other cognitive domains such as language due to time constraints. Notably, we have a more detailed neuropsychology assessment in the neuroimaging sub-study (n = 500, age 69-71) and we have previously published on the relationship between the cross-sectional nature of neuropsychology measures with brain health (Lu et al., 2021, 2020, 2019a). We will be able to assess longitudinal changes in these neuropsychological measures in due course with more testing waves. There are a high volume of tests but, in line with previous studies (Rothman, 1990), adjustments have not made for multiple comparisons and results are shown as mean difference with 95% confidence intervals at every stage to enable the reader to judge the biological importance of the results. While our sample is broadly representative of the population born in mainland Britain in 1946 (Kuh et al., 2016; Stafford et al., 2013), our findings are based on a generation of 70 year-old British-based participants who are part of a lifelong study and have higher cognition, education, SEP and self-rated health than the original cohort (James et al., 2018); so associations reported here may underestimate the strength of effects in those in the denominator population. Other limitations include treating amyloid burden as a binary metric which is a common approach but may mask small effects of cognition on amyloid burden. We do not have earlier measures of amyloid deposition and can't infer when amyloid deposition started. We are not currently able to directly characterize and address how cognitive decline is related to the biological AD spectrum as set out within the useful National Institute on Ageing and Alzheimer's Association Research (NIA-AA) framework (Jack et al., 2018). Future work will examine how cognitive trajectories are related to longitudinal changes in brain health and neuropathology.

### 4.3 Summary

In a population-based sample, subtle changes in cognitive decline of memory and processing speed across 26 years of mid- to later- life adulthood are associated with brain health indices associated with dementia at 70 years of age, including greater Aβ deposition; greater presumed cerebral small vessel disease; and smaller brain and hippocampal volume. Our findings are consistent with the idea that prodromal cognitive effects are detectable in early older-age and can be indicative of early pathology associated with dementia. Capturing and monitoring decline of cognitive domains in mid to later life is important to provide indicators of neuropathological changes, which might help identify those at risk.

## Contributors

MR, DK, NCF and JMS conceived the original study. JMN provided statistical support. CAL, TP, AK, SMB, SEK, HMS, AW, KL recruited and tested participants. DMC, IBM, CHS, MM and WC performed imaging processing and quality control. SNJ and JMN performed the analysis and drafted the initial manuscript. All authors contributed to revision, interpretation and editing of the manuscript.

## Disclosure statement

Sarah-Naomi James – Reports no disclosures, Jennifer M. Nicholas – Reports no disclosures, Kirsty Lu – Reports no disclosures, Thomas D. Parker - Supported by a Wellcome Trust Clinical Research Fellowship (200109/Z/15/Z)., Christopher A. Lane – Reports no disclosures, Ashvini Keshavan – Supported by a Wolfson Foundation Clinical Research Fellowship., Sarah E. Keuss – Reports no disclosures, Sarah M. Buchanan – Reports no disclosures, Heidi Murray-Smith – Reports no disclosures, David M. Cash – Supported by the UK Dementia Research Institute which receives its funding from DRI Ltd, funded by the UK Medical Research Council, Alzheimer’s Society and Alzheimer’s Research UK (ARUK‐PG2017‐1946),  the UCL/UCLH NIHR Biomedical Research Centre, and the UKRI Innovation Scholars: Data Science Training in Health and Bioscience (MR/V03863X/1)) - Carole H. Sudre - Supported by an MRC platform grant (EP/M020533/1) and an Alzheimer's Society Junior Fellowship (AS-JF-17-011)., Josephine Barnes – Supported by a Senior ARUK fellowship., Ian B. Malone – Reports no disclosures, Will Coath – Reports no disclosures, Marc Modat – Supported by the Leonard Wolfson Experimental Neurology Centre and an Alzheimer's Society Project Grant (AS-PG-15-025)., Andrew Wong – Reports no disclosures, Diana Kuh – Reports no disclosures, Sebastien Ourselin – Reports no disclosures, Sebastian J. Crutch - Supported by an Alzheimer's Research UK Senior Research Fellowship (ARUK-SRF2013-8)., Nick C. Fox - supported by the UCL/UCLH NIHR Biomedical Research Centre, Leonard Wolfson Experimental Neurology Centre, and the UK Dementia Research Institute at UCL., Marcus Richards – Reports no disclosures, Jonathan M. Schott - supported by the UCL/UCLH NIHR Biomedical Research Centre, UCL Hospitals Biomedical Research Centre, and Leonard Wolfson Experimental Neurology Centre. Acknowledges the EPSRC (EP/J020990/1) and European Union's Horizon 2020 research and innovation programme (Grant 666992).

## Appendix Supplementary materials

Image, application 1
