# Supplementary material for: Adulthood cognitive trajectories over 26 years and brain health at 70 years of age: findings from the 1946 British Birth Cohort
Source: Neurobiol Aging. 2023 Feb;122:22–32. doi: 10.1016/j.neurobiolaging.2022.10.003 (PMC10564626; doi:10.1016/j.neurobiolaging.2022.10.003)
Supplement: Supplementary file 1 [file mmc1.docx]

# Adulthood cognitive trajectories over 26 years and brain health at 70 years of age: Findings from the 1946 British Birth Cohort

Supplementary Appendix

# A.1 Supplementary Methods

**Study inclusion criteria**

To capitalise on the life course data and to avoid *a priori* decisions as to who might be at risk of cognitive decline, entry criteria for Insight 46 from the wider Medical Research Council National Survey of Health and Development (NSHD) were based on maximising the life course data available for analysis. The recruitment protocol (1) and an overview of the recruitment and predictors of recruitment in our sample have previously been described (2). Participants were defined as eligible for recruitment to Insight 46 if they met the criteria of having a defined set of life course data available (outlined in Table A.1 as replicated from the protocol paper (1)) and expressed willingness to come to a London-based clinic visit (overview outlined in Figure A.1 as replicated from the recruitment overview paper (2)). As demonstrated in Figure A.1, of the 841 participants invited, 502 (60%) attended the clinic; 204 (24%) refused, 28 (3%) temporarily refused, 12 (1%) did not respond, 3 (0.4%) died, 23 (3%) cancelled visits and 69 (8%) were excluded for reasons including severe claustrophobia (n = 34) and metal implants (n = 28).

Given that the NSHD has remained broadly representative of the general population (3) we were previously able to publish an extensive analysis of how sociodemographic factors, health conditions and health-related behaviours predicted recruitment and participation into Insight 46, the neuroimaging sub-study (2). In brief we found that higher educational attainment and non-manual socio-economic position (SEP) were consistent predictors of recruitment. Health-related variables were also predictive at every level of recruitment; in particular higher cognition, not smoking and better self-rating health (2). Sex and APOE-e4 status were not predictors of participation at any level. Understanding the factors that influence recruitment are important when interpreting results. Given that those at highest risk for negative outcomes may be under-represented in Insight 46, it is likely that health-related outcomes and life course risks will under-estimate those seen in the general population (2).

**Table A1.1: Original criteria of set of life course data required for Insight 46 eligibility *.**

| 1. Attendance at a clinic visit at age 60–64. |
| --- |
| 1. Parental socioeconomic position: at least one indicator of occupational social class or education |
| 1. Cognition: memory and processing speed from the 60–64 year collection AND at least one set of measures at either ages 8, 11 or 15 |
| 1. Early physical growth trajectories: birth weight and at least one measure of height and weight at ages 4–15. |
| 1. Educational attainment: highest qualification by age 26. |
| 1. Mental health: teacher ratings of behaviour and temperament at ages 13 or 15, and at least one measure of affective symptoms at ages 36, 43, 53 or 60–64. |
| 1. Blood pressure, lung function, adult height and weight: at least one measure of each at ages 36, 43, 53 or 60–64. |
| 1. Health behaviours: at least one measure of smoking and physical exercise at ages 36, 43, 53 or 60–64. |
| 1. Blood: either age 53 or 60–64 samples. |

* Replicated from our protocol paper: (1). When recruitment was underway the criteria were relaxed to remove the requirement for a measure of lung function, smoking or physical exercise (n=62).

**Figure A1.1: Deriving the sample for Insight 46.** Replicated from recruitment paper (2).

**
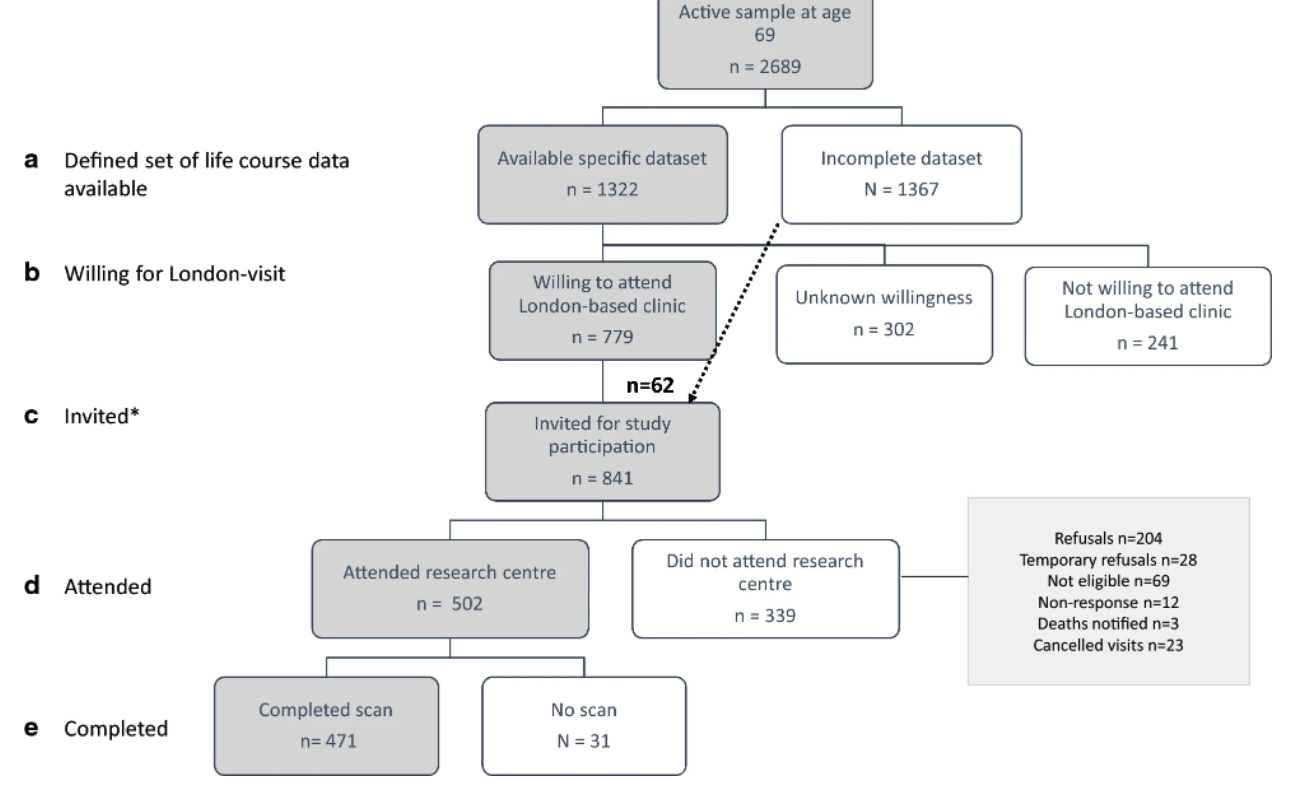
**

**Imaging protocol**

**MRI** (1, 4)**:** Imaging was performed on a single Biograph mMR 3T PET/MRI scanner (Siemens Healthcare, Erlangen), with simultaneous acquisition of dynamic PET/MR data including volumetric (1.1mm isotropic) T1 and FLAIR sequences; the full imaging protocol has been described previously (1). MRI sequences included: three-dimensional T1-weighted MPRAGE images (voxel size 1.1x1.1x1.1 mm^3^ isotropic; TE/TR = 2.92/2000, total time = 5 minutes 6 seconds) and three-dimensional FLAIR images using an IR-SPACE acquisition scheme (voxel size 1.1 x1.1x1.1 mm^3^ isotropic; TE/TR = 402/5000, total time = 6 minutes 27 seconds) (1). All MRI data were preprocessed for gradwarp and image inhomogeneity. Furthermore, all MRI data underwent a detailed quality control process by trained assessors, in line with protocols developed for commercial trials, who assess motion, coverage and other issues. T1 scans were also assessed for blurring, image wrap-around and contrast problems, and FLAIR for adequate CSF suppression (1).

Pre-processing of structural (T1, T2, FLAIR) images was carried out by applying a correction for gradient non-linearity followed by brain-masked (by registration of MNI template to the scan) N4-bias correction. An automated multi-region parcellation of the T1 images was carried out using geodesic information flow (GIF) (1).

**Amyloid PET global SUVR calculation:** GIF v.32 region of interests (ROIs) were selected to match as closely as possible the Freesurfer (version 4·5·0, surfer.nmr.mgh.harvard.edu/) composite ROI used in Alzheimer’s Disease Neuroimaging Initiative (ADNI), which includes the lateral and medial frontal, anterior and posterior cingulate, lateral parietal, and lateral temporal regions. (5).

**Brain volume:** Volumetric T1-weighted images underwent visual QC before processing using automated pipelines for whole-brain segmentation using Multi-Atlas Propagation and Segmentation (1)(6).

**Hippocampal volume:** Volumetric T1-weighted images underwent visual QC before processing using automated pipelines for hippocampal region segmentation using Similarity and Truth Estimation for Propagated segmentations followed by manual checking and appropritae editing (7).

**Bayesian Model Selection (BaMoS) white matter hyperintensity (WMH) segmentation:** BaMoS, an unsupervised automated algorithm, is based on a novel adaptive framework for the modelling of data outliers using a multivariate Gaussian mixture model4 that has been shown to perform well against other freely-available automated segmentation methods for segmentation of age-related WMH (8). Volumetric FLAIR and T1-weighted images were used for analysis. Prior to WMH segmentation, scans were reviewed by a consultant neuroradiologist, and any potential reportable findings flagged for review, according to pre-defined and published criteria. Pre-processing involved rigid registration of subjects’ FLAIR image to the T1 volumetric space using the NiftyReg opensource package, followed by brain masking. Intensity data were log transformed and corrected for intensity inhomogeneities using an additive model of bias field as a linear combination of spatially varying polynomial functions. The data are modelled hierarchically by separating the data into an inlier and an outlier part. Each part is symmetrically and jointly modelled as a mixture of multiple anatomical classes, with each one of these classes modelled as a combination of Gaussian distributions. Because the number of Gaussians necessary to characterise each tissue class is not known a priori, a split and merge strategy dynamically modifies the model structure and enables a more comprehensive investigation of the data space. After convergence of each newly suggested model using an expectation-maximisation algorithm, the Bayesian Information Criterion (BIC) is used to decide whether to accept or reject this new stage. Such criterion enforces appropriate balance between model fit and complexity. Spatial constraints are introduced through anatomical probabilistic atlases while smoothness of the segmentation is enforced by the application of a Markov Random Field. After model convergence, a post-processing step was applied in order to select candidate lesion voxels based on intensity and location rules. The formed connected components of candidate lesions were then automatically classified as lesion or artefact. The resulting probability map of WMH was then integrated to obtain the global white matter hyperintensity volume (WMHV), which included subcortical grey matter but not the infratentorial region. Validation of the BaMoS algorithm in Insight 46 was performed by comparing its performance with semi-automated segmentations, performed by two trained raters (CL and JB) on 30 Insight 46 scans. Good spatial agreement was demonstrated with both raters’ semi-automated segmentations (mean (SD) Dice coefficients 0.71 (0.11); 0.72 (0.11) respectively).

**Figure A1.2: QC for Insight 46 and available imaging metrics**

T1 QC failure (n=3)

Dementia (n=3)

Attended research centre (n=502)

Non-completion of scan (n=31): Claustrophobia (n=25)

Uncomfortable in scanner (n=3)

Concerns about radiation (n=1)

Possible metallic implant (n=1)

Study withdrawal (n=1)

PET amyloid data (n=460)

Completed phase 1 scan (n=471)

T1 brain volume data (n=468)

White matter hyperintensity volume data (n=455)

BaMoS QC failure (n=5)

Amyloid QC failure (n=8)

# A.2 Immediate WLT and delayed WLT at age 60-64 years

The word learning test (WLT) was assessed by recall of a 15-item word list where participants were shown each word for 2 seconds (Davis et al., 2017). Participants were then immediately asked to recall these words within 1 minute (immediate recall). The total number of words correctly recalled over three identical trials was summed to provide an overall score for WLT (maximum 45). Word lists alternated between study visits to minimise practice effects. Notably, the first WLT assessment in 1989 (age 43) only assessed immediate free recall and did not assess delayed recall as is commonly used with this test. The neurocognitive test battery was repeated in the same manner for the same individuals in subsequent testing waves (in 1999, 2009 and 2015) to keep consistency of measures and ability to measure intra-individual changes in these measures. However, there was an additional fourth prompt for delayed recall of the word list in the 2009 testing wave, whereby participants were asked to write down as many words as possible after another cognitive task, around 10 minutes after the initial presentation. Table A3.1 outlines a sensitivity analysis that shows there were little differences in the pattern of associations of with brain health measures depending on whether the immediate or delayed measure of WLT was used at this timepoint.

**Table A2.1: Regression analyses assessing the relationship between the word learning test immediate and delayed recall at age 60-64 years with brain health measures at age 69-71 years.**

|  | Coefficient (denoted below) | *p* | *95% CI* |
| --- | --- | --- | --- |
| **Amyloid positivity^A^** |  |  |  |
| WLT immediate recall, age 60 years | 0.96 | 0.79 | 0.71,1.29 |
| WLT delayed recall, age 60 years | 1.00 | 0.96 | 0.78,1.31 |
| **Brain volume^B^** |  |  |  |
| WLT immediate recall, age 60 years | 0.04 | 0.52 | -0.08,0.15 |
| WLT delayed recall, age 60 years | 0.04 | 0.45 | -0.14,0.06 |
| **Hippocampal volume^B^** |  |  |  |
| WLT immediate recall, age 60 years | -0.05 | 0.36 | -0.17,0.06 |
| WLT delayed recall, age 60 years | -0.03 | 0.51 | -0.13,0.07 |
| **White matter hyperintensity volume^C^** |  |  |  |
| WLT immediate recall, age 60 years | -0.07 | 0.23 | -0.19,0.04 |
| WLT delayed recall, age 60 years | -0.03 | 0.54 | -0.13,0.07 |

WLT=word learning test. All models adjusted for sex and age at scan, childhood cognitive ability, childhood and adult SEP, educational attainment and total intracranial volume (for brain, hippocampal and white matter hyperintensity volume).

^A^ A logistic regression model was conducted for amyloid status where coefficients represent an odds ratio.

^B^ Linear regression models were conducted for standardised brain volume and hippocampal volume, where coefficients represent a standardised change in standard deviation per unit change of cognition.

^C^ A generalised linear model using the gamma distribution with log link conducted for white matter hyperintensity volume where coefficients represent a relative increase.

# A.3 Different operationalising of childhood cognition

Child cognition was adjusted since this was previously shown to be associated with intercept and slope of decline in adult word learning test and search speed tests (Davis et al., 2017; Richards, 2001). Childhood cognitive ability was measured at age 8 using tests of reading comprehension, pronunciation, vocabulary and non-verbal reasoning (Pigeon, 1964)**.** Scores from each test were standardised to the tested sample at the time. In the main presented analyses, where data were missing, z-scores from assessments at age 11 or age 15 were substituted. Sensitivity analyses to investigate if the results from the main mixed effect cognitive trajectory modelling differed depending on how childhood cognition was operationalised as a covariate, using WLT trajectories and Aβ as an example, revealed very little difference in patterns (Table A2.1). Models were re-run using childhood cognition i) with and without re-standardising to the analytical sample; ii) with and without imputing missing cognitive scores with standardised scores ascertained at ages 11 and 15.

**Table A3.1: Longitudinal decline of word learning test (WLT) from age 43-69 years by Aβ status at 70 years of age. Sensitivity analyses of the main interaction term using different operationalisation metrics of childhood cognitive ability as a covariate, as listed.** All models additionally adjust for sex, age at scan, childhood and adult SEP and educational attainment.

| **Word learning test decline** | **B** | ***p*** | ***95% CI*** |
| --- | --- | --- | --- |
| *Interaction term: Interaction effect of Aβ by time per year (linear)* |  |  |  |
| Using child cognitive ability age 8, standardised to original sample (n=442) | -0.05 | **0.05** | -0.10,0.01 |
| Using child cognitive ability age 8, re-standardised to analysis sample (n=442) | -0.05 | **0.05** | -0.10,0.01 |
| Using child cognitive ability age 8, missing imputed from age 11 ability (n=462) | -0.06 | **0.01** | -0.11,-0.01 |
| Using child cognitive ability age 8, missing imputed from age 11 and 15 ability (n=466)* | -0.06 | **0.01** | -0.11,-0.01 |

*Denotes the way childhood cognitive ability is operationalised in the full main analyses.

# A.4 Interactions

To assess the potential effect modifying nature, we tested whether the relationships between the brain health measure of interest and cognitive trajectory differed by sex, APOE-*ε4* and other measured brain health measures,

# Table A4.1: P-values for the interactions terms of interest on the longitudinal decline of word learning test (WLT) and search speed measures from age 43-69 years by brain health measures at 70 years of age.

|  | Sex | Apoe-E4 | Amyloid status | BV | HV | WMHV |
| --- | --- | --- | --- | --- | --- | --- |
| **Brain health measure and cognitive trajectory of interest** | *p* | *p* | *p* | *p* | *p* | *p* |
| Amyloid status and WLT decline | 0.5 | 0.5 |  | 1.0 | 0.9 | 0.6 |
| Brain volume and search speed decline | 0.2 | 0.5 | 0.6 |  | 0.3 | 0.1 |
| Hippocampal volume and search speed decline | 0.1 | 0.2 | 0.6 | 0.3 |  | 0.5 |
| White matter hyperintensity volume and WLT decline | 0.2 | 0.5 | 0.6 | 0.1 | 0.1 |  |

WLT=word learning test; BV=brain volume; HV=hippocampal volume. WMHV=white matter hyperintensity volume.

# A.5 Associations between cognitive trajectories and brain health, with adjustments

All models were adjusted for age at scan, spanning 2 years, and known important premorbid predictors of later-life cognition and cognitive decline including sex, childhood cognition, childhood and adulthood SEP and educational attainment.

To reduce multiple testing, we selected only the cognitive trajectories and brain health measures that were significant for further testing (A._5_).

To assess the independence of associations between cognitive trajectories and specific brain measures, significant models were re-run mutually adjusting for other measured brain health measures (Aβ, brain, hippocampal and WMH volume). Models were re-run adjusting for affective mental health problems at the time of cognitive assessment to assess the potential impact of affective mood on cognitive performance and re-run excluding those who met criteria for dementia (n=3) or MCI at the time of the scan (n=7) to explore if those with cognitive impairment were driving any associations. In line with our previously published papers (Lu et al., 2019b) and based on published criteria (Petersen et al., 2013), MCI was determined as follows: 1) no clinical evidence of dementia; *and* 2) participant concern regarding cognition (memory or cognitive difficulties more than other people the same age, or if they reported that they would seek medical attention regarding their difficulties) or informant concern regarding the participant's cognition (AD8 score ≥2); *and* 3) objective evidence of either an amnestic (Logical Memory delayed recall ≥1.5 SD below the mean) or nonamnestic deficit (digit substitution score ≥1.5 SD below the mean).

**Table A5.1: Longitudinal decline of word learning test (WLT) or search speed measures from age 43-69 years of age by Aβ status, brain, hippocampal and white matter hyperintensity volume, with adjustments.**

|  | Model 0 ( sex, age at scan, childhood cognition, childhood and adult SEP, education) | | | +mutual adjustment for brain health | | | +APOE-E4 | | | | +mental health at 69 | | | | excluding MCI (n=10) | | |
| --- | --- | --- | --- | --- | --- | --- | --- | --- | --- | --- | --- | --- | --- | --- | --- | --- | --- |
|  | B | *p* | *95% CI* | B | *p* | *95% CI* | B | *p* | *95% CI* | B | | *p* | *95% CI* | B | | *p* | *95% CI* |
| **Amyloid status and WLT decline** | | | | | | |  |  |  | |  |  |  |  | |  |  |
|  | -0.06 | **0.01** | -0.11,-0.01 | -0.05 | **0.03** | -0.10,-0.00 | -0.06 | **0.01** | -0.11,-0.01 | -0.06 | | **0.02** | -0.11,-0.01 | -0.03 | | 0.14 | -0.08,0.01 |
| **Brain volume and search speed decline** | | | | | | |  |  |  | |  |  |  |  | |  |  |
|  | 0.01 | **<0.01** | 0.00,0.01 | 0.01 | **<0.01** | 0.00,0.01 | 0.01 | **<0.01** | 0.00,0.01 | 0.01 | | **<0.01** | 0.00,0.01 | 0.01 | | **0.01** | 0.00,0.01 |
| **Hippocampal volume and search speed decline** | | | | | | |  |  |  | |  |  |  |  | |  |  |
|  | 0.99 | **0.02** | 0.14,1.84 | 0.99 | **0.02** | 0.11,1.88 | 0.96 | **0.02** | 0.10,1.82 | 1 | | **0.02** | 0.15,1.86 | 0.97 | | **0.03** | 0.09,1.86 |
| **WMH volume and WLT decline** | | | | | | |  |  |  | |  |  |  |  | |  |  |
|  | -0.02 | **0.01** | -0.04,-0.01 | -0.02 | **0.01** | -0.04,-0.01 | -0.02 | **0.01** | -0.04,-0.01 | -0.02 | | **0.01** | -0.04,-0.01 | -0.02 | | **0.02** | -0.04,-0.01 |

Note: p<0.05 denoted in bold. Aβ=amyloid positivity; WLT=word learning test; CI=confidence interval; BIC=Bayesian Information Criterion; BV=brain volume; HV=hippocampal volume. WMH=white matter hyperintensity volume.

**References**

1. Lane CA, Parker TD, Cash DM, Macpherson K, Donnachie E, Murray-Smith H, *et al.* (2017): Study protocol: Insight 46 – a neuroscience sub-study of the MRC National Survey of Health and Development. *BMC Neurol*. 17: 75.

2. James S-N, Lane CA, Parker TD, Lu K, Collins JD, Murray-Smith H, *et al.* (2018): Using a birth cohort to study brain health and preclinical dementia: recruitment and participation rates in Insight 46. *BMC Res Notes*. 11: 885.

3. Stafford M, Black S, Shah I, Hardy R, Pierce M, Richards M, *et al.* (2013): Using a birth cohort to study ageing: representativeness and response rates in the National Survey of Health and Development. *Eur J Ageing*. 10: 145–157.

4. Parker TD, Cash DM, Lane CAS, Lu K, Malone IB, Nicholas JM, *et al.* (2019): Hippocampal subfield volumes and pre-clinical Alzheimer’s disease in 408 cognitively normal adults born in 1946. (Y. Huo, editor) *PLoS One*. 14: e0224030.

5. Landau SM, Mintun MA, Joshi AD, Koeppe RA, Petersen RC, Aisen PS, *et al.* (2012): Amyloid deposition, hypometabolism, and longitudinal cognitive decline. *Ann Neurol*. 72: 578–586.

6. Leung KK, Barnes J, Modat M, Ridgway GR, Bartlett JW, Fox NC, *et al.* (2011): Brain MAPS: an automated, accurate and robust brain extraction technique using a template library. *Neuroimage*. 55: 1091–108.

7. Jorge Cardoso M, Leung K, Modat M, Keihaninejad S, Cash D, Barnes J, *et al.* (2013): STEPS: Similarity and Truth Estimation for Propagated Segmentations and its application to hippocampal segmentation and brain parcelation. *Med Image Anal*. 17: 671–684.

8. Sudre CH, Cardoso MJ, Bouvy WH, Biessels GJ, Barnes J, Ourselin S (2015): Bayesian model selection for pathological neuroimaging data applied to white matter lesion segmentation. *IEEE Trans Med Imaging*. 34: 2079–102.
